# Barth syndrome without tetralinoleoyl cardiolipin deficiency: a possible ameliorated phenotype

**DOI:** 10.1007/s10545-014-9747-y

**Published:** 2014-08-12

**Authors:** Ann Bowron, Julie Honeychurch, Maggie Williams, Beverley Tsai-Goodman, Nicol Clayton, Lucy Jones, Graham J. Shortland, Shakeel A. Qureshi, Simon J. R. Heales, Colin G. Steward

**Affiliations:** 1 Department of Clinical Biochemistry, University Hospitals Bristol NHS Trust, Bristol, BS2 8HW UK; 2 NHS Barth Syndrome Service, Bristol Royal Hospital for Children, University Hospitals Bristol NHS Trust, Bristol, BS2 8BJ UK; 3 School of Cellular & Molecular Medicine, School of Medical Sciences, University Walk, Bristol, BS8 1TD UK; 4 Bristol Genetics Laboratory, North Bristol NHS Trust, Bristol, BS10 5NB UK; 5 Department of Paediatric Cardiology, Bristol Royal Hospital for Children, University Hospitals Bristol NHS Trust, Bristol, BS2 8BJ UK; 6 Department of Metabolic Disease, University Hospitals Wales, Cardiff, CF14 4XW UK; 7 Department of Paediatric Cardiology, Evelina Children’s Hospital, Guy’s and St Thomas’ NHS Foundation Trust, London, SE1 7EH UK; 8 Department of Chemical Pathology, Great Ormond Street Hospital NHS Foundation Trust, London, WC1N 3JH UK; 9 University College London Institute of Child Health, London, WC1N 1EH UK

## Abstract

Barth syndrome (BTHS) is an X-linked disorder characterised by cardiac and skeletal myopathy, growth delay, neutropenia and 3-methylglutaconic aciduria (3-MGCA). Patients have *TAZ* gene mutations which affect metabolism of cardiolipin, resulting in low tetralinoleoyl cardiolipin (CL_4_), an increase in its precursor, monolysocardiolipin (MLCL), and an increased MLCL/CL_4_ ratio. During development of a diagnostic service for BTHS, leukocyte CL_4_ was measured in 156 controls and 34 patients with genetically confirmed BTHS. A sub-group of seven subjects from three unrelated families was identified with leukocyte CL_4_ concentrations within the control range. This had led to initial false negative disease detection in two of these patients. MLCL/CL_4_ in this subgroup was lower than in other BTHS patients but higher than controls, with no overlap between the groups. *TAZ* gene mutations in these families are all predicted to be pathological. This report describes the clinical histories of these seven individuals with an atypical phenotype: some features were typical of BTHS (five have had cardiomyopathy, one family has a history of male infant deaths, three have growth delay and five have 3-MGCA) but none has persistent neutropenia, five have excellent exercise tolerance and two adults are asymptomatic. This report also emphasises the importance of measurement of MLCL/CL_4_ ratio rather than CL_4_ alone in the biochemical diagnosis of the BTHS.

## Introduction

Barth syndrome (BTHS, OMIM 3020660) is an X-linked disorder of cardiomyopathy (CM) and skeletal myopathy accompanied by neutropenia which was first described by Peter Barth and colleagues in 1983 (Barth et al 1983). Growth retardation and 3-methylglutaconic aciduria (3-MGCA) have also been described in BTHS (Kelley et al 1991), as well as a characteristic dysmorphology (Hastings et al 2009) and male fetal death resulting in miscarriage and stillbirth (Steward et al 2010).

BTHS is caused by mutations in the tafazzin (*TAZ*) gene (Bione et al 1996) which encodes proteins that belong to a family of acyltransferases known as tafazzins (Schlame et al 2002). These are involved in phospholipid biosynthesis, specifically in the incorporation of linoleic acid into phosphatidylglycerol and cardiolipin (Vreken et al 2000). Cardiolipin is a mitochondrial membrane phospholipid with a tissue-specific structure (Schlame et al 2005). The most abundant form in the mitochondria of human cardiac and skeletal muscle is tetralinoleoyl cardiolipin (CL_4_). Deficiency of CL_4_ has been identified in a range of tissues in patients with BTHS (Schlame et al 2002), as well as increased concentrations of its precursor, monolysocardiolipin (MLCL) (Houtkooper et al 2009; Valianpour et al 2002a, b). These findings have also been described in *TAZ* deficient yeast (Li et al 2007), Drosophila (Xu et al 2006) and a knock-down mouse model of BTHS (Acehan et al 2011), and are consistent with the hypothesis that the disorder is caused by defective cardiolipin remodelling.

Over 120 mutations in the *TAZ* gene have been identified, occurring in all 11 exons as well as at some splice sites (Gonzalez 2013). Most are missense mutations, small deletions or insertions, but some large exon deletions and one full gene deletion have also been described. Most are predicted to result in loss of function.

Patients with BTHS exhibit considerable phenotypic variation; the features of the disorder are variable both between patients and within individual patients over time. For example, some BTHS patients never develop CM, many have CM which ameliorates with age (Rigaud et al 2013; Roberts et al 2012; Spencer et al 2006) and up to 10 % have not been identified as being neutropenic at any stage (Clarke et al 2013). No genotype/phenotype correlations have been identified.

We describe seven patients from three families with BTHS confirmed by *TAZ* mutation analysis who have some biochemical and clinical features which differ from other BTHS cases previously described, notably lack of severe deficiency of CL_4_ in leukocytes and absence of neutropenia; two are asymptomatic adults, one of whom has never had any features of BTHS.

## Subjects and methods

### Subjects

**Fig. 1:**
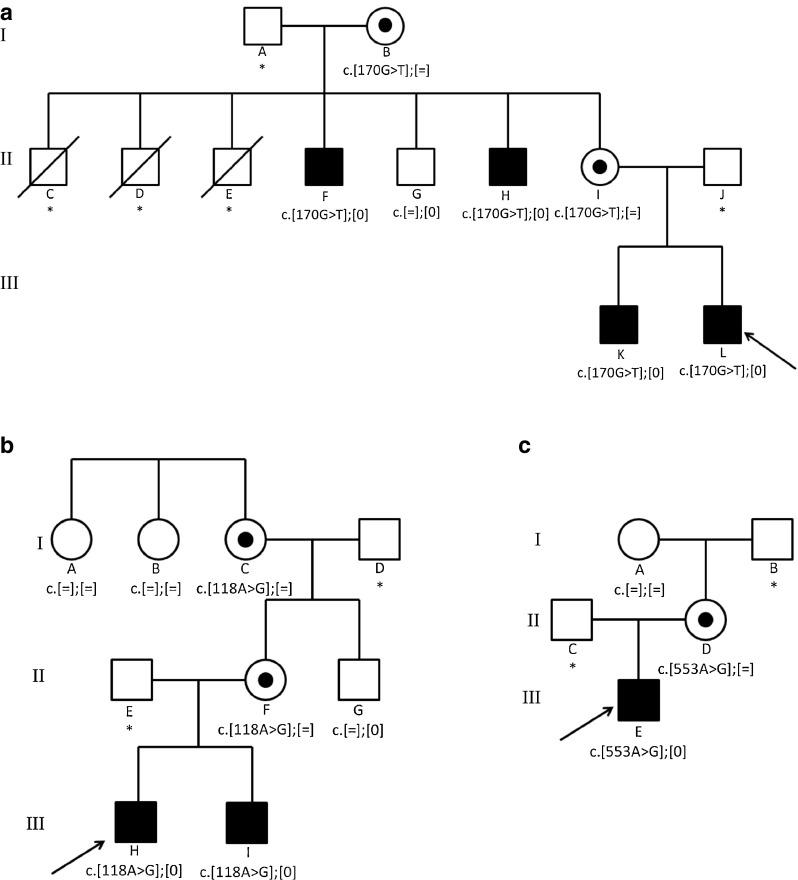
Pedigree and *TAZ* genotype of the families of subjects 1–7. Solid squares indicate males with Barth syndrome. Carrier females are represented by circles containing a black spot. * indicates patient has not been tested. Arrows indicate proband. (**a**) Subject 1 = L; subject 2 = K; subject 3 = H; subject 4 = F. (**b**) Subject 5 = H; subject 6 = I. (**c**) Subject 7 = E

Subject 1 presented in the neonatal period with hypertrophic CM and slightly increased urinary 3-MGCA. Three maternal uncles had died in infancy, indicating possible X-linked disease in the family (Fig. 1a); he was therefore investigated for BTHS. *TAZ* gene analysis identified a novel mutation in exon 2, c.170G > T (p.Arg57Leu), confirming the diagnosis. Echocardiographic appearances improved steadily and at 12 years of age are normal apart from mild left ventricular trabeculation; he is however still maintained on digoxin and ACE inhibitors. He is very active, playing competitive sports regularly including full soccer matches. His height and weight are on the 25th and 75th centiles respectively. There was one recorded episode of neutropenia (0.5 × 10^9^) in the neonatal period when he had CM, otherwise he has had no history of neutropenia and has only contracted occasional routine childhood infections.

Subject 2, the older brother of subject 1, had an uneventful neonatal course and achieved normal developmental milestones. He underwent testing for BTHS following the diagnosis of the disorder in his brother. Platelet CL_4_ was measured and reported as normal, and the diagnosis was therefore excluded. A diagnosis of BTHS was pursued subsequently with *TAZ* gene mutation analysis because of the history of early male death in this family; this identified the c.170G > T mutation in exon 2 previously identified in his brother. His cardiac function was therefore monitored and found to be normal apart from mild left ventricular trabeculation. At the age of 14 years a cardiac arrhythmia was identified which has been treated with a beta-blocker. Urine organic acid analysis has recently shown 3-MCGA. He enjoys exercise, plays team sports, cycles and is able to keep up with his peers. His height is on the 50th centile and his weight on the 25th–50th centile. He has never had an episode of recorded neutropenia.

Subject 3 is 44 years of age and is the maternal uncle of patients 1 and 2. He presented in the neonatal period with decompensated CM which responded well to treatment with digoxin for one year. His cardiac function continues to be monitored, but he is on no treatment and has had no further cardiac disease. Following the diagnosis of BTHS in his nephews, and in light of the history of early death of three of his brothers, he underwent *TAZ* gene testing and the familial mutation was identified. He has not been tested for 3-MGCA. He has normal exercise tolerance, has a physically demanding job and is 1.85 m tall with a body-mass index (BMI) of 24. He has no history of neutropenia or recurrent infections.

Subject 4 is 47 years of age, the elder brother of subject 3. His development was normal and he has no medical history of note. Echocardiography performed at the time of the diagnosis of BTHS in his nephew showed borderline septal and left ventricular hypertrophy which did not require treatment. He recently started treatment for essential hypertension. *TAZ* gene mutation analysis was performed as part of the investigation of his family for BTHS and the c.170G > T mutation found. He has not been investigated for 3-MGCA. He is very active, previously playing competitive football and has a physically demanding job. He is 1.8 m tall and has a BMI of 32. Neutropenia has not been detected.

Subject 5, from a second unrelated family (Fig. 1b), was under investigation for global developmental delay and failure to thrive when, at the age of 2 years, he presented with congestive heart failure due to dilated cardiomyopathy (DCM). A CM screen was normal apart from 3-MGCA on urine organic acid analysis. The subsequent finding of DCM in his younger brother (subject 6, described below) suggested a genetic cause and he was investigated for BTHS. A novel *TAZ* mutation, c.118A > G (p.Asn40Asp), was identified. His cardiac function improved, allowing weaning of treatment from the age of seven years; at 15 years of age his cardiac function is normal on maintenance ACE inhibitors and diuretics. However he continues to have impaired motor skills, skeletal myopathy, difficulty walking and problems with fatigue, needing to take regular breaks during physical education lessons at school. He also has significant learning difficulties. His height is on the 0.4th centile and weight on the 9th–25th centile. He has never had an episode of recorded neutropenia.

Subject 6, younger brother of subject 5, presented at 13 months of age with bronchiolitis, requiring admission to the Paediatric Intensive Care Unit (PICU). He was diagnosed with DCM and 3-MGCA was identified. Following the findings in his brother, *TAZ* mutation analysis was performed and the c.118A > G mutation identified. His CM resolved quickly and by the age of 2 years all medication was stopped. He is now 11 years old, is well, has no learning problems and has normal cardiac function. He is active, joining in physical education with his peers, but suffers fatigue after these lessons. His height and weight are on the 2nd–9th centiles. He has had no episodes of neutropenia and no history of infections.

Subject 7 from a third unrelated family (Fig. 1c), presented to his local emergency department at 4 months of age with a 2-day history of poor feeding. Chest X-ray showed cardiomegaly and echocardiogram showed a dilated left ventricle (LV) with poor contractility. He became acidotic and unwell and was transferred to PICU at a specialist paediatric centre. Further echocardiogram suggested left ventricular non-compaction. A CM screen was normal apart from a slight increase in urine 3-MGCA. The screen included the finding of normal bloodspot CL_4_. He was treated with diuretics, ACE inhibitors and a beta-blocker, resulting in improvement of LV function over the following year and an increase of fractional shortening from 5 to 35 %. His presentation was typical of other patients with BTHS seen by the clinical team so further investigations were pursued. Leukocyte MLCL/CL_4_ was measured and found to be above the reference range, prompting *TAZ* mutation analysis which identified a previously reported pathogenic mutation in exon 7, c.553A > G (p.Met185Val). His LV function has improved from his initial presentation and is stable on ACE inhibitors, digoxin and a beta-blocker. At 2 years of age he is well and active; his motor skills are developing well. His height and weight are on the 2nd–9th centile. He has had no recorded neutropenia.

### Cardiolipin analysis

Subjects 1, 2, 5 and 6 had blood samples taken on a routine visit to the NHS BTHS service (NBSS) clinic at Bristol Royal Hospital for Children (BRHC). An additional sample was taken during routine venepuncture for leukocyte MLCL/CL_4_ analysis as part of development of a diagnostic test for BTHS. MLCL/CL_4_ analysis in leukocytes was performed in subjects 3 and 4 following the findings in their nephews. Written informed consent was obtained from the parents and, where appropriate, the subjects for sample analysis to be performed. Approval for this investigation was obtained from the local NHS research ethics committee. Subject 7 had MLCL/CL_4_ measured due to a strong clinical suspicion that he had BTHS. All except subjects 3 and 4 have had cardiolipin analysis repeated on a later sample using the same method to confirm the findings.

Leukocytes were extracted from K-EDTA blood, phospholipids extracted, CL_4_ and MLCL(18:1)_2_(16:0) measured by LC-MS/MS (Bowron et al 2013) and the MLCL/CL_4_ ratio calculated. Results were compared with those obtained from 156 paediatric controls and 27 other patients with BTHS confirmed by *TAZ* mutation analysis referred to the NBSS.

### Mutation analysis

All seven patients had *TAZ* mutation analysis performed as part of their investigations for BTHS or for family studies. Consent for genetic testing was obtained at the time of sample collection.

Genomic DNA was received from an external laboratory or isolated from peripheral blood leukocytes using a Gentra Puregene cell kit (QIAGEN Ltd). Coding regions of the *TAZ* gene (11 exons including intron/exon boundaries extending to the branch sites) were amplified in ten fragments using a MegaMix (MicroZone) and GC-RICH (Roche) PCR system. Primers were designed to GenBank Reference Sequence NM_000116.3 using Primer3 software (Koressaar and Remm 2007; Untergasser et al 2012). M13 tagged bi-directional sequencing was undertaken using a BigDye Terminator v3.1 Cycle Sequencing Kit and 3730 DNA Genetic Analyzer (Applied Biosystems) with Mutation Surveyor DNA Variant Analysis Software v3.97 (Softgenetics). Alamut software v2.3.1 (Interactive Biosoftware, Rouen, France) was used to predict the effect of genetic variation. The software integrates PolyPhen-2, Align GVGD and SIFT and five splice site prediction programs: SpliceSiteFinder, MaxEntScan, Human Splice Finder, NNSPLICE and GeneSplicer.

### Full blood counts

Neutrophil counts were recorded on subjects 1–7 during routine clinic appointments; 75 neutrophil counts were obtained. These were compared with neutrophil counts measured on other BTHS patients attending the NBSS; results were excluded from samples taken after patients had begun treatment with granulocyte colony stimulating factor (G-CSF). Data was obtained from their referring hospitals and from their outpatient visits to BRHC. 652 neutrophil count results were obtained from 15 patients.

### Statistics

Data was analysed using GraphPad Prism (San Diego, California, USA). Results from different groups of patients were compared using a Mann–Whitney test with significance set at *p* < 0.05.

## Results

**Fig. 2:**
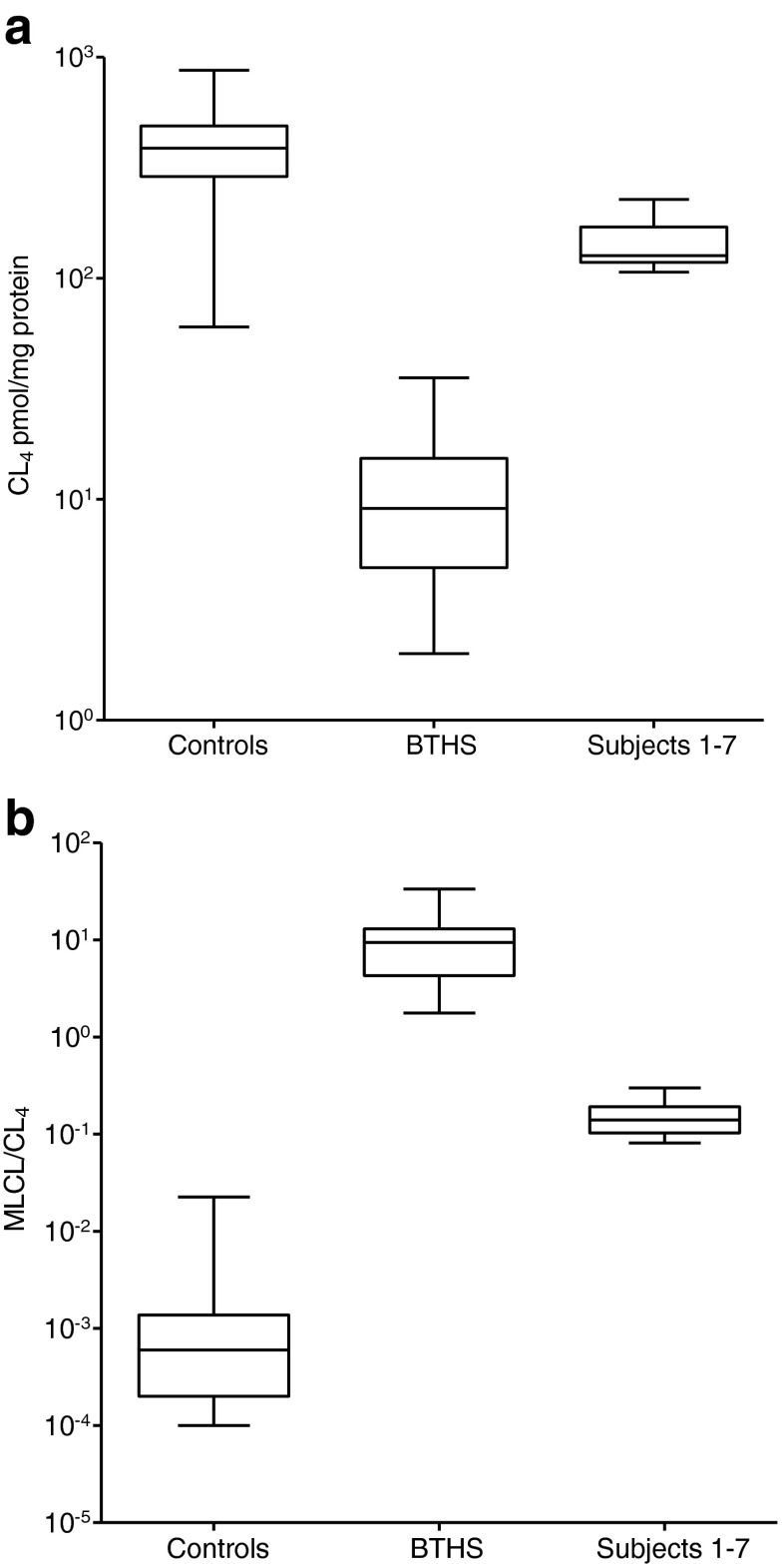
Box-and-whisker plot of results of (**a**) CL_4_ and (**b**) MLCL/CL_4_ analysis in patients 1–7 compared with controls and other patients with BTHS. The horizontal bar represents the median, the box the interquartile range and the vertical bars the range. There is a significant difference between all three groups on each graph *p* < 0.0001

Leukocyte CL_4_ results from subjects 1–7 (median 127 pmol/mg protein [range 107–227]) were significantly higher than those found in other BTHS patients (9.1 pmol/mg protein [2.0–35], *p* < 0.0001) and lower than control values (388 pmol/mg protein [60–872], *p* < 0.0001). The CL_4_ results in subjects 1–7 overlapped with the lower quartile of the control values. The MLCL/CL_4_ ratios in subjects 1–7 (0.14 [0.08–0.30]) were lower than in other BTHS patients (9.4 [1.8–33], *p* < 0.0001), but higher than controls (0.6 × 10^−4^ [1.0 × 10^−4^ − 0.02] *p* < 0.0001). There was no overlap between the three groups (Fig. 2).

**Fig. 3:**
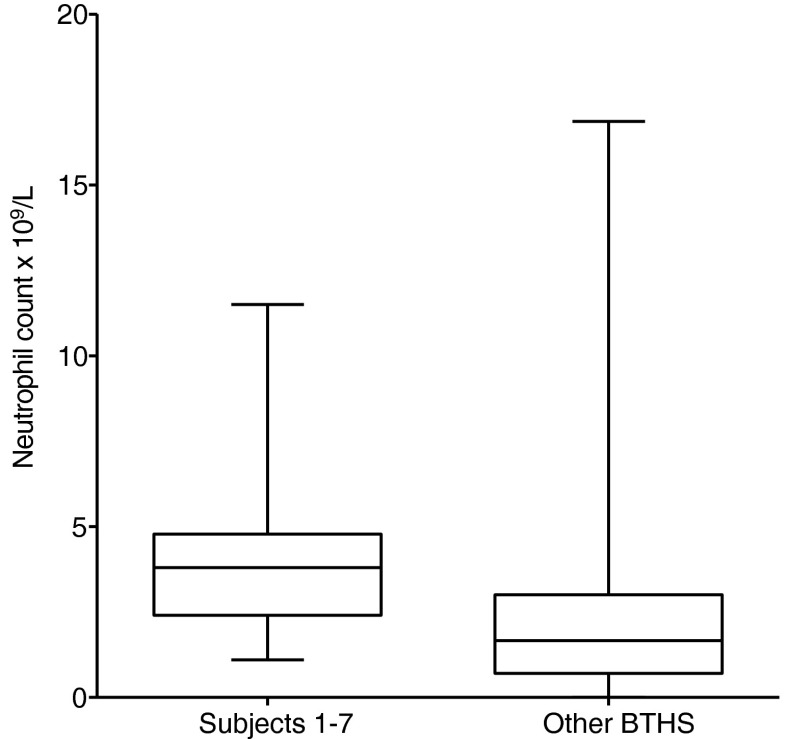
Box-and-whisker plot of neutrophil counts in patients 1–7 compared with other patients with BTHS. Results from subjects 1–7 are significantly higher than other BTHS patients *p* < 0.0001

Only one low neutrophil count has been recorded in subjects 1–7 (0.5 × 10^9^ in subject 1 during the neonatal period when he had CM). The values recorded in this cohort were significantly higher than neutrophil counts obtained from other BTHS patients (*p* < 0.0001) (Fig. 3).

## Discussion

We have described seven subjects from three families with BTHS confirmed by the finding of a mutation in the *TAZ* gene. They do not have profound CL_4_ deficiency typical of BTHS, although increased MLCL confirms that there is an abnormality in cardiolipin remodelling. We believe this to be the first description of BTHS without severe CL_4_ deficiency. In two subjects, platelet or bloodspot CL_4_ had been measured previously and found to be normal, resulting in the diagnosis of BTHS being excluded initially. This was subsequently revised due to family history or clinical features and the finding of a MLCL/CL_4_ ratio significantly above the reference range; this highlights the importance of measurement of this ratio, rather than isolated assay of CL_4_, when performing biochemical investigation of BTHS.

The mutation c.170G > T in exon 2 (p.Arg57Leu) was found in subjects 1–4. Arginine at position 57 is highly conserved and is predicted to have a strongly adverse effect on protein function (Alamut 2013) (Table 1). A yeast model of BTHS has been used to investigate biochemical function of 21 tafazzin mutations. The corresponding yeast mutation, y.Lys65Leu, demonstrated wild-type acyltransferase activity at 30 °C, but demonstrated some thermolability with reduced acyltransferase activity and increased MLCL/CL at 37 °C (Whited et al 2012). The clinical course of surviving members of this family with BTHS has been relatively mild. However it is important to note the family history. Both subjects 1 and 3 had a severe cardiac presentation in the neonatal period and three maternal uncles of the two boys died in infancy: one at four months (reportedly due to myocarditis), the second at birth following a breech delivery (the exact cause of death is unknown) and the third of *E.Coli* infection on day one of life proceeding to cardiac arrest and death from pneumonia. These deaths preceded discovery of the *TAZ* gene and development of cardiolipin analysis so BTHS cannot be confirmed, although these histories are strongly suggestive. If they did have BTHS, this demonstrates that this mutation, whilst conferring relatively mild disease in the four subjects described here, may have a severe disease course, possibly under different environmental challenges or in combination with other gene variants. These findings emphasise the wide phenotypic variation in BTHS, even within the same family.

**Table 1:** *In silico* comparison of *TAZ* mutations found in cases 1–7 with other previously reported *TAZ* mutations causing a classic Barth syndrome phenotype. Also included is a variant of unknown significance in the same region that was found by NHLB1 Exome Sequencing Project with a frequency of 1/10,560

Mutation	Exon	Nucleotide conservation	Amino acid conservation (considering 12 species)	Protein Domain	AGVD	Grantham	SIFT	PolyPhen-2	Splicing	Cardiolipin	Phenotype
c.118A > G, p.(Asn40Asp)	2	Moderately conserved	Highly conserved	Not listed in Alamut. Phospholipid domain appears to start at codon 41	C15	23	Affect protein function (score 0.00)	HumDiv- Possibly damaging 0.651 HumVar - Benign 0.272	n/a	See subjects 5&6	See subjects 5&6
c.149 T > C, p.(Leu50Pro)	2	Moderately conserved	Highly conserved	Phospholipid/glycerol acyltransferase	C35	98	Affect protein function (score 0.00)	HumDiv- Probably damaging 1.00 HumVar - Probably damaging 0.999	n/a	Low CL4, increased MLCL/CL4	Cardio-myopathy, neutropenia, FTT, 3MGCA
c.170G > T, p.(Arg57Leu)	2	Moderately conserved	Highly conserved	Phospholipid/glycerol acyltransferase	C0	102	Affect protein function (score 0.01)	HumDiv- Probably damaging 0.998 HumVar - Probably damaging 0.997	n/a	See subjects 1-4	See subjects 1-4
c.253C > T, p.(Arg85Cys) Variant of Unknown Significance	3	Weakly conserved	Moderately conserved	Phospholipid/glycerol acyltransferase	C15	180	Affect protein function (score 0.01)	HumDiv- Probably damaging 0.990 HumVar - Possibly damaging 0.745	n/a	Found on EVS	Found on EVS (EurAm 0.01 %)
c.281G > A p.(Arg94His)	3	Moderately conserved	Highly conserved	Phospholipid/glycerol acyltransferase & Tafazzin	C25	29	Affect protein function (score 0.00)	HumDiv- Probably damaging 0.999 HumVar - Probably damaging 0.945	n/a	Low CL4, increased MLCL/CL4	Dilated cardio-myopathy & neutropenia
c.553A > G, p.(Met185Val)	7	Moderately conserved	Moderately conserved	Phospholipid/glycerol acyltransferase	n/a	n/a	n/a	n/a	New acceptor site created, confirmed by functional studies	See subject 7	See subject 7

AGVD (Align GVGD) combines the biophysical characteristics of amino acids and protein multiple sequence alignments to predict where missense substitutions in genes of interest fall in a spectrum from enriched deleterious to enriched neutral. Align GVGD is an extension of the original Grantham difference to multiple sequence alignments and true simultaneous multiple comparisons. Its scores are divided into seven classes, class C65 is for those most likely to interfere with protein function and class C0 the least likely

PolyPhen-2 considers evolutionary conservation, physiochemical differences and the proximity of the substitution to predicted functional domains and/or structural features. The tool presents a confidence score ranging from 0.0 to 1.0

Prediction categories: *probably damaging* — it is with high confidence supposed to affect protein function or structure; *possibly damaging* — it is supposed to affect protein function or structure; *benign* — most likely lacking any phenotypic effect; *unknown* — in some rare cases, the lack of data do not allow PolyPhen2 to make a prediction

The HumDiv (human mutation/divergency training set), and HumVar (human polymorphic variants training set) and the multiple sequence alignment. HumDiv is the preferred model for evaluating rare alleles, dense mapping regions identified by genome-wide association studies and analysis of natural selection. HumVar is the preferred model for diagnostics of Mendelian diseases which requires distinguishing mutations with drastic effects from all the remaining human variation, including abundant mildly deleterious alleles. It is advisable to record both, as ideally they should come up with the same classification, although they can have different confidence scores

EVS: Exome Variant Server (NHLBI 2014)

The *TAZ* variant found in subjects 5 and 6, c.118A > G (p.Asn40Asp), is at a highly conserved amino acid position and is predicted to have an adverse effect on the protein using SIFT *in silico* analysis; although another tool, PolyPhen-2, is inconclusive with its pathogenicity prediction (Alamut 2013) (Table 1). The corresponding yeast mutation, y.Asn48Asp, has been reported to have identical biochemical activity to wild type yeast at both 30 °C and 37 °C, indicating that it has no effect on yeast tafazzin function (Whited et al 2012). However, as there is no population frequency data available for this variant it is unlikely to be a benign polymorphism. These two boys both had severe cardiac disease in early childhood accompanied by 3-MGCA, indicating that this *TAZ* variant has pathological effects.

**Fig. 4:**
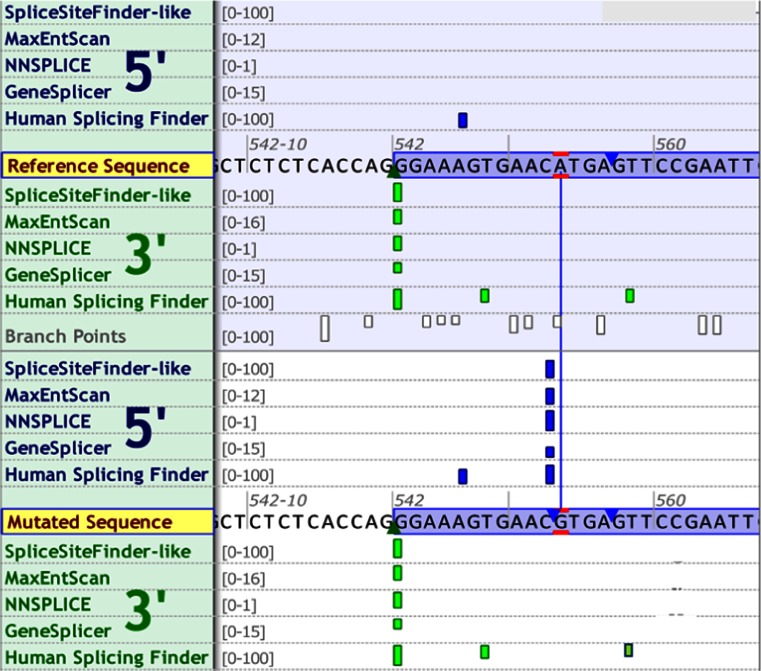
*In silico* analysis of the *TAZ* c.553A > G variant found in patient 7 using various splicing prediction programs (Alamut v2.3.1). Top section pale blue shaded area is wild-type sequence. Bottom section is mutated sequence. Dark blue bars represent predicted donor splice sites. Green bars represent predicted acceptor splice sites. The diagram shows the creation of a new strong donor splice site at the variant position

The mutation in subject 7, an exon 7 variant c.553A > G, predicts the substitution p.Met185Val. Bioinformatics tools indicate that this has an adverse effect on protein function, generating a new splice donor signal within exon 7 (Alamut 2013) (Fig. 4, Table 1). Functional RNA analysis proved that this mutation causes retention of intron 6 and partial deletion of exon 7, confirming pathogenicity (r.[541 + 1_542-1 ins; r.553_583del]; p.Lys182Glnfs*4) (Fan et al 2013). The mutation has not been modelled in yeast. Fan et al recently described a patient with this mutation; he had DCM requiring cardiac transplantation, neutropenia (0.5 × 10^9^/L) treated with G-CSF and an increased lymphoblast cell line MLCL/CL ratio consistent with BTHS.

Urine organic acid analysis was performed in five of this group of subjects and 3-MGCA found in each on at least two occasions. It is known that 3-MGCA is an inconsistent feature of BTHS (Rigaud et al 2013; Schmidt et al 2004; Wortmann et al 2012) although the mechanism for this organic aciduria is not known. The patient described by Fan et al had no 3-MGCA on multiple urine organic acids analyses, whereas subject 7 in our study with the same mutation had 3-MGCA identified on at least three occasions on samples measured in two different laboratories. The variation in 3-MGCA between two unrelated patients with the same *TAZ* gene mutation indicates that 3-MGCA excretion has other influences which are not understood.

BTHS patients attending the NBSS exhibit characteristic slow linear growth with a trend of increasing adiposity throughout adolescence (Clarke et al 2013). Subjects 5–7 have height and weight consistent with this. Whilst subjects 1 and 2 are taller than many BTHS patients, their heights fall within the range seen in our clinic.

The clinical phenotype of this group is therefore relatively mild and atypical for BTHS. Only one of this group suffers from skeletal myopathy. Exercise tolerance is excellent in five and hard to assess due to young age and concomitant CM in a sixth. Subjects 1–4 have been able to take part in full competitive sport. Two of the subjects have no history of CM. Finally, none of the BTHS subjects described in this report has chronic neutropenia or recurrent infection as a feature of their disease.

In conclusion, this report demonstrates that some *TAZ* mutations do not result in profound CL_4_ deficiency and highlights the importance of measurement of MLCL/CL_4_ rather than CL_4_ alone in the biochemical diagnosis of BTHS. In this group of subjects without severe CL_4_ deficiency, neutrophil counts are significantly higher than in other BTHS subjects and the disease course so far is relatively mild, suggesting a possible ameliorated form of BTHS and a correlation between biochemistry and phenotype. Further investigations into the function of these mutations and the resulting biochemical and clinical phenotype may help to elucidate the role of tafazzin and the pathogenesis of this complex multi-system disorder.

## References

[CR1] Acehan D, Vaz F, Houtkooper RH (2011). Cardiac and skeletal muscle defects in a mouse model of human Barth syndrome. J Biol Chem.

[CR2] Alamut (2013) Software 2.3.1 incorporating PolyPhen-2, SIFT and multiple site prediction tools. http://www.interactive-biosoftware.com

[CR3] Barth PG, Scholte HR, Berden JA (1983). An X-linked mitochondrial disease affecting cardiac-muscle, skeletal-muscle and neutrophil leukocytes. J Neurol Sci.

[CR4] Bione S, Dadamo P, Maestrini E, Gedeon AK, Bolhuis PA, Toniolo D (1996). A novel X-linked gene, G4.5. is responsible for Barth syndrome. Nat Genet.

[CR5] Bowron A, Frost R, Powers VC, Thomas P, Heales SR, Steward C (2013). Diagnosis of Barth syndrome using a novel LC-MS/MS method for leukocyte cardiolipin analysis. J Inherit Metab Dis.

[CR6] Clarke S, Bowron A, Gonzalez I (2013). Barth syndrome. Orphanet J Rare Dis.

[CR7] Fan Y, Steller J, Gonzalez IL (2013). A Novel Exonic Splicing Mutation in the TAZ (G4.5) Gene in a case with atypical Barth syndrome. JIMD Reports.

[CR8] Gonzalez I (2013) Human *Tafazzin (TAZ)* Gene Mutation and Variation Database. http://www.barthsyndrome.org

[CR9] Hastings R, Steward CG, Tsai-Goodman B, Newbury-Ecob RA (2009). Dysmorphology of Barth syndrome. Clin Dysmorphol.

[CR10] Houtkooper RH, Rodenburg RJ, Thiels C (2009). Cardiolipin and monolysocardiolipin analysis in fibroblasts, lymphocytes, and tissues using high-performance liquid chromatography-mass spectrometry as a diagnostic test for Barth syndrome. Anal Biochem.

[CR11] Kelley RI, Cheatham JP, Clark BJ (1991). X-linked dilated cardiomyopathy with neutropenia, growth retardation and 3-methylglutaconic acid. J Pediatr.

[CR12] Koressaar T, Remm M (2007). Enhancements and modifications of primer design program Primer3. Bioinformatics.

[CR13] Li GL, Chen SL, Thompson MN, Greenberg ML (2007). New insights into the regulation of cardiolipin biosynthesis in yeast: implications for Barth syndrome. Biochim Biophys Acta-Mol Cell Biol Lipids.

[CR14] NHLBI (2014) Exome Sequencing Project Exome variant Server. NIH Heart, Lung and Blood Institute. http://evs.gs.washington.edu/EVS/

[CR15] Rigaud C, Lebre A-S, Touraine R (2013). Natural history of Barth syndrome: a national cohort study of 22 patients. Orphanet J Rare Dis.

[CR16] Roberts AE, Nixon C, Steward CG (2012). The Barth Syndrome Registry: distinguishing disease characteristics and growth data from a longitudinal study. Am J Med Genet A.

[CR17] Schlame M, Towbin JA, Heerdt PM, Jehle R, DiMauro S, Blanck TJJ (2002). Deficiency of tetralinoleoyl-cardiolipin in Barth syndrome. Ann Neurol.

[CR18] Schlame M, Ren MD, Xu Y, Greenberg ML, Haller I (2005). Molecular symmetry in mitochondrial cardiolipins. Chem Phys Lipids.

[CR19] Schmidt MR, Birkebaek N, Gonzalez I, Sunde L (2004). Barth syndrome without 3-methylglutaconic aciduria. Acta Paediatr.

[CR20] Spencer CT, Bryant RM, Day J (2006). Cardiac and clinical phenotype in Barth syndrome. Pediatrics.

[CR21] Steward CG, Newbury-Ecob RA, Hastings R (2010). Barth syndrome: an X-linked cause of fetal cardiomyopathy and stillbirth. Prenat Diagn.

[CR22] Untergasser A, Cutcutache I, Koressaar T et al (2012) Primer3-new capabilities and interfaces. Nucleic Acids Res 40 10.1093/nar/gks596 PMC3424584 22730293

[CR23] Valianpour F, Wanders RJA, Barth PG, van Overmars H, Gennip AH (2002). Quantitative and compositional study of cardiolipin in platelets by electrospray ionization mass spectrometry: application for the identification of Barth syndrome patients. Clin Chem.

[CR24] Valianpour F, Wanders RJA, Overmars H (2002). Cardiolipin deficiency in X-linked cardioskeletal myopathy and neutropenia (Barth syndrome, MIM 302060): a study in cultured skin fibroblasts. J Pediatr.

[CR25] Vreken P, Valianpour F, Nijtmans LG (2000). Defective remodeling of cardiolipin and phosphatidylglycerol in Barth syndrome. Biochem Biophys Res Commun.

[CR26] Whited K, Baile MG, Currier P Claypool SM (2012) Seven functional classes of Barth syndrome mutation. Hum Mol Genet 10.1093/hmg/dds447 PMC3606006 23100323

[CR27] Wortmann SB, Kluijtmans LA, Engelke UFH, Wevers RA, Morava E (2012). The 3-methylglutaconic acidurias: what’s new?. J Inherit Metab Dis.

[CR28] Xu Y, Condell M, Plesken H (2006). A Drosophila model of Barth syndrome. Proc Natl Acad Sci U S A.

